# Dr. Clark on Ipecacuanha Enema in Dysentery

**Published:** 1812-08

**Authors:** Tho. Clark

**Affiliations:** No. 17, Denmark-street, Soho


I have the honor to be, Gentlemen,

Your most obedient and humble Servant,

JAMES LITLE, Surgeon,

*His Majesty's Ship Salvador del Mundo,*
*at Plymouth, June* 5, 1812.

---

*To the Editors of the Medical and Physical Journal.*

GENTLEMEN,

YOU possibly may remember, that in my small publication of August 1801, on Diseases of the Army, &c. I have mentioned that I first prescribed decoctions of ipecacuanha as injections in dysentery, at Columbe, in the island of Ceylon, in the year 1797, when I was surgeon of his Majesty's 19th regiment of foot.

It

It affords me much pleasure to state, that, during my residence in France for upwards of these last nine years, I have had many opportunities of observing the good effects of ipecacuanha decoctions, not only in dysentery, but also in internal piles, and in flatulent distension of the bowels, from whatever cause they may have arisen.

While at Verdun in France, I likewise received a very satisfactory account concerning the effects of the same medicine from Mr. Connin, surgeon of the navy, who was made prisoner of war in the Mediterranean. He asserted to me, that during last war, while on the same station, he lost many men of dysentery; but that, during the present war, although he had the charge of a considerable number of sick laboring under dysentery, yet he did not lose one patient. This astonishing difference of success he attributes chiefly to ipecacuanha injections.

Since my return to London, I also have obtained very satisfactory additional information on the same subject from my friend, Mr. Archibald Barklimore, surgeon, of High-street, Bloomsbury.

In a letter from our mutual friend, Mr. Baird, surgeon in the Hon. East-India Company's service, to Mr. Barklimore, dated Tigris, off the Cape of Good Hope, 18th May, 1810, the following passage is contained: "With regard to professional news, little has occurred in our ship to afford opportunity for observation. I cannot help mentioning, however, (because it gives me much pleasure,) that a case has lately occurred in which the valuable remedy that our friend Clark communicated to us has been very successful in curing dysentery. One of the ship's company, who had been afflicted with dysentery for several weeks, and who had been much reduced by mercury, (which seemed to be the cause of the disease,) was snatched from the jaws of death by the ipecacuanha given in clysters. Mr. Graham, surgeon of the ship, consulted me when he had lost all hopes of his patient's recovery. At this time his evacuations were generally slimy, mixed with blood, and he sometimes passed liquid stools of the color of coffee. I recommended the ipecacuanha, and, though my hopes of its being beneficial were by no means sanguine, I was delighted to find that the poor man was soon relieved by it. I expect to do much good with it in India."

Another letter to Mr. Barklimore from Mr. Baird, dated Port Louis, Isle of France, 5th Dec. 1810, contains the following observations: "I was attached to the 22d regiment, and embarked with Colonel Kelso, in the Illustrious, 74, with a detachment of 175 men. We had a long passage, and were sickly. Dysentery made dreadful ravages amongst the ship's company. Nearly 70 men died of it in nine weeks. I was very fortunate in only losing three, and two of these were old bad cases. I used Clark's remedy freely with much advantage."

In a third letter from Mr. Baird to Mr. Barklimore, dated Port Louis, Mauritius, 10th Nov. 1811, the subsequent remarks are made: "I have mentioned to you before how successful I had found Clark's mode of treating dysentery. I have daily opportunities of confirming it. Most of my fellow-labourers have been in a situation to judge of its effects, and have all, I believe, adopted it. I did not fail on these occasions to do him the justice of quoting him as the teacher of the practice. A few days ago I went into a bookseller's shop here, and, in rummaging over a parcel of medical books, his little work presented itself to my agreeable surprise. I was not long of buying it, you may be sure. I have derived the greatest pleasure from a perusal of it, and shall certainly consider myself indebted to him for much valuable practical information."

Hence it would seem that the use of decoctions of ipecacuanha in dysentery will soon become general. It would therefore be highly gratifying to me, and probably very beneficial to mankind, if your readers who may have prescribed the remedy in question would acquaint the world at large, through the medium of your valuable Medical Journal, with the effects produced by it under their directions.

I here think proper to mention, for the information of those who have not seen my publication of 1811, that the form of decoction which I found most successful in adults, was about three drams of ipecacuanha boiled in a quart of water down to a pint, strained, and given all at once as a lavement. In cases of internal piles, for reasons that must appear obvious to every one, it is in most instances unnecessary to administer more than half a pint of the medicine at a time.

I have the honor to be, Gentlemen,

With the utmost respect,

Your most obedient Servant,

*No.* 17, *Denmark-street, Soho,* THO. CLARK, M.D.

*June* 17, 1812.

